# SOCS1, a novel interaction partner of p53 controlling oncogene-induced senescence

**DOI:** 10.18632/aging.100163

**Published:** 2010-06-26

**Authors:** Frédérick A. Mallette, Viviane Calabrese, Subburaj Ilangumaran, Gerardo Ferbeyre

**Affiliations:** ^1^ Département de Biochimie, Université de Montréal, Montréal, Québec H3C 3J7, Canada; ^2^ Present address: Terry Fox Molecular Oncology Group and the Bloomfield Center for Research on Aging, Sir Mortimer B Davis Jewish General Hospital, Lady Davis Institute for Medical Research, Montréal, Québec H3T 1E2, Canada; ^3^ Immunology Division, Department of Pediatrics; Faculty of Medicine and Health Sciences, University of Sherbrooke, Sherbrooke, Canada

**Keywords:** SOCS1, senescence, p53, ATM, ATR, STAT5, cytokines

## Abstract

Members of the signal transducers and activators of transcription (STATs) family of proteins, which connect cytokine signaling to activation of transcription, are frequently activated in human cancers. Suppressors of cytokine signaling (SOCS) are transcriptional targets of activated STAT proteins that negatively control STAT signaling. SOCS1 expression is silenced in multiple human cancers suggesting a tumor suppressor role for this protein. However, SOCS1 not only regulates STAT signaling but can also localize to the nucleus and directly interact with the p53 tumor suppressor through its central SH2 domain. Furthermore, SOCS1 contributes to p53 activation and phosphorylation on serine 15 by forming a ternary complex with ATM or ATR. Through this mechanism SOCS1 regulates the process of oncogene-induced senescence, which is a very important tumor suppressor response. A mutant SOCS1 lacking the SOCS box cannot interact with ATM/ATR, stimulate p53 or induce the senescence phenotype, suggesting that the SOCS box recruits DNA damage activated kinases to its interaction partners bound to its SH2 domain. Proteomic analysis of SOCS1 interaction partners revealed other potential targets of SOCS1 in the DNA damage response. These newly discovered functions of SOCS1 help to explain the increased susceptibility of *Socs1* null mice to develop cancer as well as their propensity to develop autoimmune diseases. Consistently, we found that mice lacking SOCS1 displayed defects in the regulation of p53 target genes including Mdm2, Pmp22, PUMA and Gadd45a. The involvement of SOCS1 in p53 activation and the DNA damage response defines a novel tumor suppressor pathway and intervention point for future cancer therapeutics.

## SOCS1, cancer and senescence

Cytokines are secreted proteins that regulate different cellular processes including survival, proliferation and differentiation. Following binding to their receptors, cytokines activate the Janus kinases (JAK1, JAK2, JAK3 and Tyk2) leading to the phosphorylation of tyrosine residues on the cytoplasmic portion of the receptor creating docking sites for signaling molecules containing a SH2 domain [1,2]. Members of the STAT family of proteins that are recruited to the phosphorylated cytokine receptors themselves become phosphorylation substrates for JAK kinases. Phosphorylated STAT proteins homo- or hetero- dimerize and translocate to the nucleus to activate transcription of target genes by binding to specific response elements in their promoter regions. Among these cytokine-induced proteins, members of the SOCS family constitute important negative regulators of the JAK/STAT signaling pathway.

**Figure 1.**
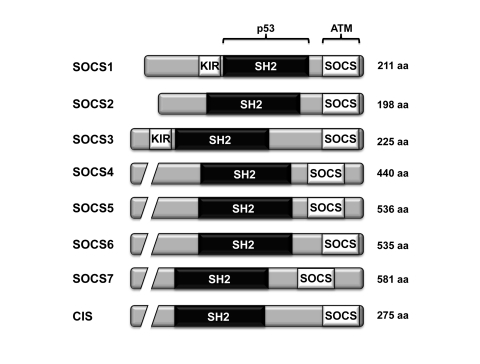
The domain architecture of the different members of the SOCS family of proteins. All eight members of the SOCS family harbor a central SH2 domain and a C-terminal SOCS box. Both SOCS1 and SOCS3 also contain a kinase inhibitory region (KIR). The region of SOCS1 interacting with p53 and ATM are shown [34].

There are eight members of the SOCS family of proteins (CIS, SOCS1-7), each of which harbor a central SH2 domain and a C-terminal SOCS box region [3] (Figure 1). The suppressor of cytokine signaling SOCS1 was initially identified as a cytokine-inducible inhibitor of STAT signaling [4,5,6].  Through its SH2 domain, SOCS1 can directly bind phosphorylated JAK2 to prevent the phosphorylation of STAT. SOCS1 also possesses a kinase inhibitory region (KIR), a domain composed of less than 30 amino acids, which shares homology with the pseudosubstrate inhibitory region of JAK and leads to inhibition of the catalytic activity of JAK [7,8]. The SOCS box allows recruitment of elongin B/C and Cullin 2 to form an ubiquitin E3 ligase complex [9,10].  This allows the SOCS protein to operate as an adaptor to trigger ubiquitination and degradation of proteins involved in cellular signaling including JAK [11], TEL-JAK2 [12], IRS-1/2 [13], FAK [14], Vav [15] and Mal [16]. It is currently thought that SOCS1 contributes to tumor suppression due to its ability to control and terminate the activation of STATs [17,18,19,20,21,22,23,24,25]. On the other hand, the relationship between SOCS1 and other tumor suppressor pathways and the cellular mechanisms by which SOCS1 might exert its tumor suppression remain largely unexplored.

To prevent the formation of cancer, normal cells possess intrinsic tumor suppressor mechanisms that are triggered upon oncogene activation. Like apoptosis, cellular senescence opposes cellular transformation by limiting the proliferation of cells expressing oncogenes. In normal human diploid cells, oncogene activation causes a permanent growth arrest with features of cellular senescence [26]. We have recently extended the list of oncogenes known to trigger the senescence response to include the JAK/STAT5 pathway. The transcription factor STAT5 is implicated in tumor formation by regulating important cellular processes including cell cycle progression, apoptosis, angiogenesis and metastasis [27]. However, in normal cells, expression of Tel/Jak2 or constitutively activated allele of STAT5A and B initiated a cell cycle arrest in G1 associated with markers of premature cellular senescence and activation of the tumor suppressors Rb and p53 [28,29,30].

## SOCS box proteins and the regulation of p53

 The activation of the p53 pathway following oncogene activation is crucial to induce senescence in normal cells. In mice, stimulation of p53 is dependent on p19ARF (Alternative Reading Frame), which is induced by several oncogenes [31,32]. However, the role of ARF in oncogene-induced senescence in human cells is still unclear [33]. In order to identify new regulators of p53 activation following constitutively activated STAT5 expression in normal cells, we performed microarray analysis covering the entire human transcriptome. We observed that the expression of SOCS1 was highly increased at both mRNA and protein level during STAT5-induced senescence [34]. Unexpectedly, SOCS1 expression in normal human fibroblasts was sufficient to trigger a p53-dependent cell cycle arrest displaying features of the senescence phenotype. This function of SOCS1 was dependent on the integrity of its SOCS box. In addition, SOCS1, but not a mutant lacking the SOCS box domain, led to the accumulation of phosphorylated p53 on serine 15 and increased transcription of the p53 target gene p21CIP. The knockdown of SOCS1 during STAT5-induced senescence reduced the phosphorylation of p53 on Ser15, diminished the nuclear accumulation of p53 and compromised the development of senescence phenotype [34]. The remaining activated p53 and partial bypass of the senescence response observed following the knockdown of SOCS1 might arise from the ability of STAT5 to engage multiple signaling pathways to ensure p53 activation. For example, STAT5 can directly transactivate the promoter of the PML gene and stimulate its expression in a p53-independent fashion [30].  The PML protein can then inhibit Mdm2 and stimulate p53 [35,36] contributing to the senescence phenotype [37,38].

SOCS1 mediated STAT5-induced senescence via an unexpected protein-protein interaction between the SH2 domain of SOCS1 and the transactivation domain of p53 [34]. Because the transactivation domain of p53 harbors no tyrosine residues, the binding should occur independently of tyrosine phosphorylation, as reported before for SOCS1 binding to Vav [15] and for other SH2 domains as well [39,40]. The von Hippel-Lindau protein (VHL), another SOCS box-containing protein, has been recently shown to interact with p53. This interaction does not rely on an SH2 domain but on the SOCS box domain of VHL. However, like SOCS1, VHL facilitates p53 interaction with the DNA damage activated kinase ATM [41]. Hence, SOCS1 links DNA damage signals stimulated by oncogenic activity to p53.

Interestingly, SOCS1 is not the only protein inhibitor of STAT implicated in the regulation of p53 activity. The protein inhibitors of activated STAT, PIAS1 and PIASy both promote the sumoylation and transcriptional activity of p53 [42,43,44]. However, the mechanism of activation of p53 by PIAS is still unclear. While the sumoylation of p53 by PIAS1 has been demonstrated [43], a mutated PIAS1 lacking the RING finger-like domain and defective in promoting p53 sumoylation was sufficient to activate p53 [44]. Furthermore, by controlling the activity of both p53 and Rb, PIASy regulates Ras-induced senescence and apoptosis [42]. These data suggest that the control of STAT signaling is tightly linked to the activation of p53 to possibly control the JAK/STAT oncogenic pathway.

## Inhibitors of STATs activity and the DNA damage response

The stimulation of p53 during oncogene-induced senescence is associated with the activation of the DNA damage response [28,45,46]. The DNA damage observed in normal cells expressing activated oncogenes may be due to reactive oxygen species [47] and/or some type of replicative stress [45,46]. SOCS1-induced senescence was accompanied by the activation of the DNA damage-regulated kinases ATM and Chk2. Since the stimulation of p53 reporters by SOCS1 was partially blocked in cells depleted of ATM, ATM might participate in the SOCS1-dependent activation of p53. Using pulldown assays, we demonstrated that SOCS1 interacted with both ATM and ATR through its SOCS box (Figure 1) [34]. ATM is an important mediator of the senescence response by activating the p53 pathway, mainly through phosphorylation of the Ser 15 residue [28,45,46]. Depletion of SOCS1 during STAT5-induced senescence caused a dramatic decrease in Ser15 phosphorylation of p53. In order to form a ternary complex with p53 and ATM, SOCS1 must localize to the nucleus. We confirmed that SOCS1 is able to localize to the nucleus and that endogenous SOCS1 colocalized to DNA damage foci with ATM during STAT5-induced senescence [34], thus reinforcing the notion that SOCS1 is a mediator of the DNA damage response. Not only SOCS1 but also other proteins controlling JAK/STAT signaling are known to localize to DNA damage sites. PIAS1 and PIAS4 were also shown to localize to DNA breaks and contribute to the DNA damage response by sumoylating BRCA1 [48,49].  Together, these findings strongly suggest a close link between cytokine signaling and the DNA damage response.

**Figure 2.**
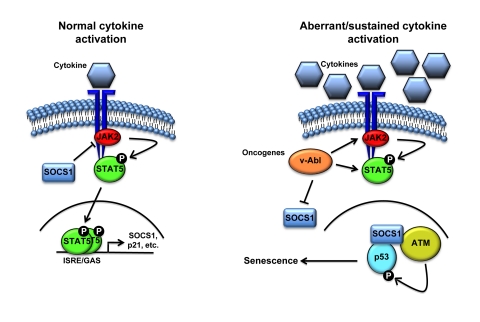
Schematic representation of the cell proliferation control exerted by SOCS1. Following activation of the receptor by cytokine binding, JAK phosphorylates the receptor creating a docking site for STATs. JAK then phopshorylates STATs causing its release from the receptor, allowing dimerization and translocation to the nucleus to activate the transcription of specific genes including members of the SOCS family. Subsequently, SOCS terminates cytokine signaling by blocking JAK activity and STAT recruitment to the receptor. However, aberrant activation of STAT5 triggered by oncogenic fusion kinases like TEL-JAK2 might result in sustained levels of SOCS1 that can activate p53 by forming a complex with ATM and p53.

## Cytokines, senescence and SOCS1: an emergency switch to control proliferation

Senescent cells secrete numerous cytokines and other mediators that modify the tissue microenvironment. The sum of these secreted factors constitutes what has been named the senescence-associated secretory phenotype (SASP) [50]. Among the SASP factors, IL-6 is required for the oncogene-induced senescence and induction of the tumor suppressor p15INK4B [51]. Furthermore, persistent, but not transient, DNA damage signaling triggers the ATM-dependent IL-6 secretion, presumably to call attention to the presence of damaged cells [52]. During oncogene-induced senescence, IL-6 also amplifies the secretion of IL-8 [51], which with GROαactivates the CXCR2 receptor to reinforce senescence [53]. Among the factors secreted by senescent cells, IGFBP7 [54] and PAI-1 [55] contribute to the growth arrest response, while p53 regulates expression of chemokines directing the immune system to permit the clearance of senescent cells [56].  Collectively, these reports suggest that cytokine signaling could prevent tumor formation by promoting cellular senescence.

The capacity of SOCS1 to activate the p53 pathway can establish an emergency anti-proliferative program in cells exposed to sustain or aberrant cytokine stimulation (Figure 2). Following normal activation of the JAK/STAT pathway, SOCS1 blocks the phosphorylation of STAT by inhibiting or degrading JAK2. However, aberrant and sustained stimulation of STAT might induce a molecular switch allowing SOCS1 to localize to DNA breaks and stimulate ATM-dependent activation of p53.

## A general role for SOCS1 in the DNA damage response

The localization of SOCS1 to DNA breaks during STAT5-induced senescence raises numerous questions. First, does the SOCS1 ubiquitin ligase activity contribute to the DNA damage response? A novel cascade of ubiquitination controlled by the E3 ubiquitin ligases RNF8/RNF168 and HERC2 have recently been reported to control the recruitment of BRCA1 and 53BP1 by ubiquitinating the histones H2A and H2AX [57,58,59,60,61,62]. The presence of SOCS1 at DNA breaks could not only regulate ATM-mediated p53 activation but also control the DNA repair process. Second, what are the mechanisms underlying the nuclear transport of SOCS1 and its presence at DNA damage foci? Since most of its interacting partners were localized to the plasma membrane, SOCS1 was considered to be mostly a cytoplasmic protein, but recent evidences suggest that it can localize to the nucleus under certain conditions including STAT5-induced senescence [34,63]. A bipartite nuclear localization signal (NLS) located between the SH2 domain and the SOCS box allows nuclear localization of SOCS1 [63,64]. However, the mechanism controlling the active transport of SOCS1 remains unclear. A clearer understanding of the mechanisms controlling SOCS1 nuclear localization would be crucial to determine how SOCS1 mediates its tumor suppressor activity. Post-translational modifications like ubiquitination and phosphorylation that have been shown to control the nuclear localization of p53 [65,66,67] and STAT [68] could also control the nucleo-cytoplasmic shuttling of SOCS1. Exclusion of SOCS1 from the nucleus would prevent the formation of the ternary complex with p53 and ATM, preventing the activation of p53. Furthermore, the phosphorylation status of SOCS1 could regulate its activity since aberrant SOCS1 phosphorylation is associated with cellular transformation. Actually, phosphorylation of SOCS1 triggered by the oncogenic v-Abl kinase impedes the SOCS1-Elongin B/C interaction, leading to sustained JAK/STAT signaling [69]. v-Abl signaling induces multiple serine/threonine kinases including members of the Pim kinase family. Pim-1 and Pim-2 are required for efficient cellular transformation mediated by v-Abl [70] and are able to phosphorylate SOCS1 and disrupt its binding to Elongin C [71]. Because SOCS1 requires the SOCS box to form a complex with ATM, v-Abl- or Pim kinase-mediated phosphorylation could potentially interfere with this interaction and block p53 activation. Therefore, it appears that aberrant phosphorylation by oncogenic kinases could interfere with the tumor suppressor activities of SOCS1 by at least two different mechanisms: phosphorylated SOCS1 would not be able to inhibit the JAK/STAT pathway and to interact with ATM and promote p53 activation.

**Table I.** Identification of SOCS1 interaction partners by mass spectrometry*.

**Protein **	**Function**
Elongin C	Interacts with SOCS box [10]
Elongin B	Interacts with SOCS box [10]
Pericentrin	Cells depleted of pericentrin enter senescence due to p53 activation [72].
SHC (Src homology 2 domain containing) transforming protein 1 (**SHC1**)	Member of the Shc protein family of molecular adaptors, SHC1 promotes apoptosis by its redox activity. SHC1 is implicated in the control of oxidative stress and life span in mammals [73].
Tripartite motif-containing 28 (**TRIM28** or **KAP1**)	TRIM28 is implicated in transcriptional control through its interaction with the Kruppel-associated box repression domain. TRIM28 contributes to DNA repair mechanisms [74].
5'-nucleotidase, cytosolic II (**NT5C2**)	NT5C2 hydrolyzes 5-prime-monophosphate (IMP) and other purine nucleotides. NT5C2 is implicated in the maintenance of a constant composition of intracellular purine/pyrimidine nucleotides [75].
BCL2-associated transcription factor 1 (**BCLAF1**)	BCLAF1, a transcriptional repressor that interacts with members of the BCL2 family of proteins, promotes apoptosis [76].
Human positive cofactor 4 (**PC4**)	Suppressor of oxidative mutator phenotype [77]. Accumulates at DNA damage foci [78].

Finally, the role of SOCS1 as a mediator facilitating the interactions of ATM and ATR with their targets suggests that other interaction partners of SOCS1 could also become the substrates of ATM/ATR-dependent phosphorylation during the DNA damage response. Proteomic analysis of SOCS1 complexes revealed putative interactions with several proteins that play a role in the DNA damage response, apoptosis or oxidative stress pathways (Table I). Future work will determine which functions of SOCS1 apply to every one of its interaction partners: ubiquitination followed by proteolytic degradation or DNA damage stimulated phosphorylation.

## Conclusions

Studies on molecular mechanisms underlying cellular senescence have made significant contributions to the discovery of novel regulators of tumor suppressor pathways. Using microarrays or cDNA / siRNA screens, multiple researchers have identified novel regulators of p53 or Rb in controlling tumor formation. Using this approach to study STAT5-induced senescence, we identified SOCS1 as an important activator of the p53 and the DNA damage response. Surprisingly, the SOCS box represents a binding motif for ATM and ATR [34]. To date, about 40 proteins are known to harbor a SOCS box domain. Clearly further work will determine whether SOCS box-containing proteins also participate in the DNA damage response and control oncogenesis.
